# Urogenital schistosomiasis and soil-transmitted helminthiasis (STH) in Cameroon: An epidemiological update at Barombi Mbo and Barombi Kotto crater lakes assessing prospects for intensified control interventions

**DOI:** 10.1186/s40249-017-0264-8

**Published:** 2017-02-27

**Authors:** Suzy J. Campbell, J. Russell Stothard, Faye O’Halloran, Deborah Sankey, Timothy Durant, Dieudonné Eloundou Ombede, Gwladys Djomkam Chuinteu, Bonnie L. Webster, Lucas Cunningham, E. James LaCourse, Louis-Albert Tchuem-Tchuenté

**Affiliations:** 1 Department of Parasitology, Liverpool School of Tropical Medicine, Pembroke Place, Liverpool, L3 5QA UK; 2 Centre for Schistosomiasis and Parasitology, Yaoundé, Cameroon; 3 Department of Life Sciences, Parasites and Vectors Division, Natural History Museum, London, SW7 5BD UK; 4 Laboratory of Parasitology and Ecology, Faculty of Sciences, University of Yaoundé I, Yaoundé, Cameroon; 5 National Programme for the Control of Schistosomiasis and Intestinal Helminthiasis, Ministry of Public Health, Yaoundé, Cameroon; 6 London Centre for Neglected Tropical Disease Research, London, UK

**Keywords:** *Schistosoma haematobium*, *Strongyloides*, Female genital schistosomiasis, WASH, *Bulinus*, *Indoplanorbis exustus*

## Abstract

**Background:**

The crater lakes of Barombi Mbo and Barombi Kotto are well-known transmission foci of schistosomiasis and soil-transmitted helminthiasis having had several important control initiatives previously. To collect contemporary epidemiological information, a cross-sectional survey was undertaken inclusive of: signs and symptoms of disease, individual treatment histories, local water, sanitation and hygiene (WASH)-related factors and malacological surveillance, with molecular characterisation of specimens.

**Methods:**

At each lake, a community cross-sectional survey was undertaken using a combination of stool and urine parasitological sampling, and interview with pro-forma questionnaires. A total of 338 children and adults participated. Material from snail and parasite species were characterised by DNA methods.

**Results:**

Egg-patent prevalence of urogenital schistosomiasis was 8.7% at Barombi Mbo (all light-intensity infections) and 40.1% at Barombi Kotto (21.2% heavy-intensity infections). Intestinal schistosomiasis was absent. At Barombi Kotto, significantly more women reported signs and symptoms associated with female genital schistosomiasis. While there had been extensive recent improvement in WASH-related infrastructure at Barombi Mbo, water contact risk scores were higher among schistosomiasis-infected participants (*P* < 0.001) and at Barombi Kotto in general (*P* < 0.001). Across both lakes, mean prevalence of STH was very low (6.3%) evidencing an impressive decrease of 79.0% over the last decade; neither *Strongyloides stercoralis* nor *Ascaris lumbricoides* were found. A total of 29 freshwater sampling sites were inspected for snails, 13 in Barombi Mbo and 16 in Barombi Kotto; water chemistry differed significantly (*P* < 0.0001) between lakes for both mean pH (7.9 *v.* 9.6) and mean conductivity (64.3 μS v. 202.1 μS) respectively. Only two *Bulinus camerunensis* found on the central island of Barombi Kotto were observed to shed schistosome cercariae, but schistosome DNA was later detected in *Bulinus* sampled from both lakes as well as in *Indoplanorbis exustus*, an invasive species from Asia.

**Conclusions:**

STH is currently at very low levels while urogenital schistosomiasis is of greatest concern at Barombi Kotto. This assessment highlights a unique opportunity for further study of the epidemiological dynamics at these crater lakes, to evaluate future intensified interventions both in terms of gaining and sustaining control at Barombi Kotto or in moving towards local interruption of transmission of both diseases at Barombi Mbo.

**Electronic supplementary material:**

The online version of this article (doi:10.1186/s40249-017-0264-8) contains supplementary material, which is available to authorized users.

## Multilingual abstracts

Please see Additional file 1 for translations of the abstract into five official working languages of the United Nations.

## Background

There are several neglected tropical diseases (NTDs) endemic throughout Cameroon that pose a significant threat to the well-being of the populace, estimated to be just over 20 million people [1]. Coordinated at the national level by the Ministry of Public Health, there are ongoing interventions against NTDs, especially those amenable to preventive chemotherapy, such as trachoma [2], lymphatic filariasis [3] and onchocerciasis [4]. With regard to schistosomiasis and soil-transmitted helminthiasis (STH), previous mapping initiatives have described the distributions of diseases [5–12]. It has been identified that more than two million people have schistosomiasis infection, and a further five million live in high transmission areas within the country [13]. The focus of efforts today is upon integrated mass drug administration (MDA) with praziquantel and mebendazole, primarily targeted to children of school-age along with education and social mobilisation [13]; where resources have permitted, inter-sectoral actions have improved water, sanitation and hygiene (WASH) factors at a variety of locations [14–17].

**Fig. 1:**
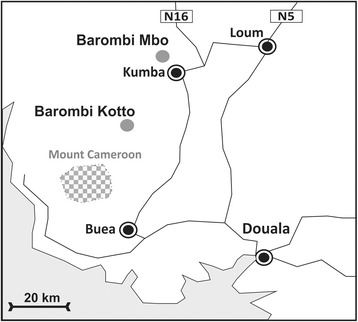
Sketch map of South West Cameroon with the crater lakes of Barombi Mbo and Barombi Kotto depicted

Three species of human schistosome exist in Cameroon, with some unique features that include cross-specific interactions between *Schistosoma mansoni* and *Schistosoma haematobium* [18], variable performance of praziquantel treatment at mixed transmission foci [19] and hybridization between *Sc. haematobium* and *Schistosoma guineensis* [20]*.* The competitive exclusion of the latter species [21] has been the subject of long-term studies in the field [22–24] and in the laboratory [20, 25, 26]. Furthermore, multidisciplinary studies on the transmission of urogenital schistosomiasis have also included medical malacology [27–30] as well as implementation of local snail control with focal molluscicides [31]. This was pioneered by Brian Duke and Peter Moore some 40 years ago [32–34] in the crater lakes Barombi Mbo and Barombi Kotto, located near Kumba in South-West, Cameroon (see Fig. 1) in seminal attempts to control urogenital schistosomiasis [35]. The prevalence of egg-patent infection with *Sc. haematobium* at these lakes was originally reported by Zahra in 1953 to be 91.0% Barombi Mbo and 76.0% Barombi Kotto [36].

Soil-transmitted helminths are also endemic in Cameroon; a study from 2010 revealed an overall STH prevalence of 46.2% in the South-West Region, with trichuriasis having highest prevalence (32.9%), followed by ascariasis (24.3%) and hookworm (15.0%) [37, 38]. The distribution of other STHs such as threadworm (*Strongyloides* spp.) remain poorly described. However, *Strongyloides stercoralis* has been noted within a HIV-cohort [39] and in children within peri-urban areas of Kumba [40]; an area also noted for autochthonous cases of paragonimiasis. A related zoonotic threadworm, *Strongyloides fulleborni* has been found in helminthological surveys of forest monkeys of the genus *Cercopithecus*, animals either eaten as bush meat or raised as semi-domestic pets [41].

In November 2015, a multidisciplinary implementation research network entitled COUNTDOWN was formed to investigate and support the scale-up of interventions against NTDs [42]. On 9^th^ October 2015, The COUNTDOWN programme was officially launched in Yaoundé by the Minister of Public Health, His Excellency André Mama Fouda [43]. Where appropriate, the programme is developing national strategies to expand preventive chemotherapy against schistosomiasis and STH, alongside assessing future opportunities to interrupt transmission locally. Given the historical significance of the crater lakes Barombi Mbo and Barombi Kotto [27, 32–34, 44], a contemporary situational comparison was undertaken at each lake, referencing previous parasitological data from in 2004, specifically with the aim of providing an epidemiological update at each lake. The three complementary objectives were to: (i) describe the prevalence and intensity of schistosomiasis (*Sc. mansoni* and *Sc. haematobium* infections) and STH (inclusive of *St. stercoralis*) in each community, (ii) assess disease associations with demography, reported morbidity and water, sanitation and hygiene (WASH) and (iii) document the distribution, abundance and infection status of encountered snails, alongside molecular characterisation of snails and schistosomes. Taken as a whole, this analysis will enable assessment of prospects for more intensified disease-control interventions and prioritisation of resources for further study.

### Study design and methods

This cross-sectional study was conducted during June 2016 at Barombi Mbo (4°40′N 9°24′E) and Barombi Kotto (4°28′N 9°16′E), South-West Cameroon. As population census data were not available by village, a preliminary sample size (*n* = 270 across both sites) was based on an estimated prevalence of 50% from a survey near Kumba [15]; the sample sizes were then recalculated based on assessed population sizes in the field and local knowledge of differences in STH prevalence at each site (expected to be 50% versus 5% at either crater lake). A target sample size of 169 participants from Barombi Kotto and 87 from Barombi Mbo was set, powered to 95% with 5% precision.

### Ethical approval and individual consent

Study protocols were approved by the Liverpool School of Tropical Medicine Research Ethics Committee (M1516-18 and M1516-06) and the Cameroon National Ethical Committee of Research for Human Health. Participation involved obtaining informed consent initiated at a variety of levels following a process of sequential community meetings explaining the purpose of the study and its methods, thereafter obtaining written consent from adults and guardians of children. All participants found infected with schistosomiasis or STH were offered praziquantel (40 mg/kg) and (or) mebendazole (500 mg) treatment.

### Assessment of schistosomiasis and soil-transmitted helminth infection

A single stool and mid-morning urine sample was each collected from all participants. Urine was first inspected visually for macrohaematuria according to a colour reference and turbidity chart [45] before being tested with reagent strips, Siemens Multistix® 10 SG and Siemens CLINITEK® Microalbumin 9 reagent dipstick, using a CLINITEK Status® + autoanalyser (Siemens Healthcare Ltd, Camberley, Surrey, UK) which have been used previously for detailed urological assessments [46] and a urine-CCA dipstick (Rapid Medical Diagnostics®, Pretoria, South Africa) for detection of intestinal schistosomiasis. For each urine sample, up to 60 ml but at least 10 ml, was filtered by plastic syringe across a 25 μm nylon pore filter within a 2.5 cm diameter swinnex filter holder. To visualise eggs, nylon filters were stained with Lugol’s iodine then counted under the microscopy at x100 magnification, with egg tallies expressed per 10 ml urine filtered [47]. Stool samples underwent four separate stool tests undertaken in parallel; I) a faecal occult blood test was performed [48], II) a double thick Kato-Katz smear was prepared for each sample and viewed at x100 magnification [48], III) approximately 5 g of stool was inspected by the Baermann concentration method, incubated for 2 h, following centrifugation and inspection of faecal pellet [47] and IV) a charcoal stool culture was prepared with approximately 3 g of stool and later incubated at ambient temperature inside a cooler box for 72 h before inspection under the dissecting microscope for parasite larvae [47].

### Participant questionnaire

A pro forma questionnaire was implemented (*available upon request from JRS*), used in a face-to-face interview setting to record each participant’s demographics, general signs and symptoms, anthelminthic treatment history as well as access and utilization of local WASH-related infrastructure. Urological symptoms included self-reported dysuria, macrohaematuria, frequency of urination, lower abdominal pain and lower back pain. Frequency of each symptom was recorded as never occurring, or occurring daily, weekly or monthly. An additional set of questions was directed to females ≥16 years of age to investigate female genital schistosomiasis (FGS). FGS symptomatology included questions pertaining to intermenstrual bleeding, genital itching, vaginal discharge, dyspareunia and post coital bleeding.

Local water and sanitation facilities were inspected by the field team and classified into improved and unimproved facilities, according to definitions from the WHO/UNICEF Joint Monitoring Programme for Water Supply and Sanitation (JMP) (WHO & UNICEF, 2015). A water contact risk score was calculated, based on that of Rudge et al*.* [49] by pooling affirmative responses to questions such as swimming, fishing, bathing etc., whereby water exposure activities were categorised by frequency of occurrence (never, daily, weekly or monthly), being assigned a categorical weighting [49].

### Malacological surveys

Shoreline sites to be surveyed were based upon convenience sampling through observation of human water contact sites and by having adequate accessibility by foot or by canoe for the field team. At each site, four collectors searched by hand and with metal collection sieves for 20 min, recording all aquatic snails encountered. Global positioning system (GPS) coordinates, altitude and location photographs were taken with an Oregon 650 receiver (Garmin, Olathe, Kansas, USA). Water temperature (°C), pH and conductivity (μS) were recorded with a HI-98129 Pocket EC/TDS and pH Tester (Hanna Instruments Ltd, Leighton Buzzard, Bedfordshire, UK)*.* All collected snails of medical importance were initially identified according to morphological keys of David S. Brown [50]. Snails were counted, graded by placement against ruler into small, medium and large size categories for each species, then transferred into plastic cups containing mineral water. Snails were subsequently exposed to natural or artificial light for two hours, the water was decanted and viewed under a dissecting microscope for cercariae and identified following Frandsen and Christensen [51].

### Molecular characterisation of snails and schistosomes

After inspection for cercariae, snails were then placed into absolute ethanol before transfer to the UK where genomic DNA was obtained according to standard extraction protocols [52]. Genetic variation within a partial region of the mitochondrial cytochrome oxidase sub-unit 1 (*cox*1) gene [53], following modified protocols described by Kane et al*.* [54], was obtained. Snails were also assayed for presence of schistosome DNA by real-time PCR with Taqman® probes using schistosome–specific primers described by Verweij [55]. In total, 18 schistosome miracidia hatched from infected urine were placed on Whatman® FTA® indicator cards (Sigma-Aldrich, Gillingham, Dorset, UK), then inspected for sequence variation within the ribosomal internal transcribed spacer (ITS) and mitochondrial *cox*1 using standard methods and bioinformatics protocols [56, 57].

### Data analysis

All numerical data were double-entered using Microsoft Excel 2013 and checked, with tabulations, graphs and statistical analyses conducted using IBM Statistical Package for the Social Sciences (IBM SPSS Statistics for Windows, Version 22.0. Armonk, NY: IBM Corp.). Egg counts and intensity of infection are well known to follow non-parametric distributions. Other variables were investigated as to whether they were distributed normally; parametric and non-parametric tests were then used accordingly. Helminth prevalence was calculated with 95% binomial confidence intervals. Chi-square and Fisher’s exact tests were used to check for associations between helminth infection, site, risk factors and morbidity. Mann–Whitney U-tests were used to check for differences in intensity of infection and water contact risk score by site. Univariable analysis and multivariable logistic regression analysis was conducted for *Sc. haematobium* associations with independent variables. Age group (preschool-aged children (PSAC); school-aged children (SAC); adults), gender and village site were included as core variables in all regression models, and a stepwise regression approach was used to arrive at the most parsimonious adjusted model, applying a 5% level of statistical significance.

## Results

In total, 338 participants were recruited to the study across both villages, 126 at Barombi Mbo and 212 at Barombi Kotto; post-hoc calculations using observed prevalence indicated that sample sizes were exceeded for both communities for all diseases. The mean age across the study population was 22.5 years (from 1.0 to 83.5 years; Table 1). Of the adult cohort, 84 (55.6%) participants were male and 67 (44.4%) were female. Three women (2.1%) were pregnant and five (5.2%) reported to be menstruating and not formally included in analysis of haematuria.

**Table 1:** Number of participants by study site and cohort age with egg-patent prevalence of urogenital schistosomiasis

		Barombi Mbo (*n* = 126)	Barombi Kotto (*n* = 212)	Total (*n* = 338)
Adults >16 years Prevalence	Population *n* (%)	77 (61.1)	74 (34.9)	151 (44.7)
	Mean age (range) *n* (%) [95% CI]	39.0 (16.5-83.2) 9 (11.7) [5.0-21.5]	42.9 (16.1-83.5) 31 (41.9) [33.0-56.9]	40.9 (16.1-83.5) 40 (26.5) [21.4-37.0]
SAC 6 ≤ 16 years Prevalence	Population *n* (%)	33 (26.2)	79 (37.3)	112 (33.1)
	Mean age (range) *n* (%) [95% CI]	9.6 (6.3-15.9) 1 (3.0) [0.0-9.3]	10.9 (6.5-16.0) 40 (50.6) [40.0-62.6]	10.5 (6.3-16.0) 41 (36.6) [28.1-46.5]
PSAC ≤6 years Prevalence	Population *n* (%)	16 (12.7)	59 (27.8)	75 (22.2)
	Mean age (range) *n* (%) [95% CI]	3.8 (1.5-4.5) 0 (0.0) [0.0-12.0]	3.7 (1.0-6.0) 14 (23.7) [13.2-36.0]	3.7 (1.0-6.0) 14 (18.7) [10.1-28.8]

*SAC* school-aged children, *PSAC* pre-school aged children

### Prevalence and intensity of schistosomiasis

Prevalence of schistosomiasis is shown in Table 1 across the different demographic groups. All *Sc. haematobium* infections at Barombi Mbo were classified as light (arithmetic mean egg count of all infected cases 2.2 eggs per 10 ml). At Barombi Kotto however, of 85 people infected (40.1% of total population), 18 infections (21.2% of those with egg-patent infection) were classified as heavy (greater than 50 eggs per 10 ml) with arithmetic mean egg count of all infected cases being 50.5 eggs per 10 ml. This difference between sites was significant (*P* < 0.001). At Barombi Mbo intensity of infection was highest among adults while at Barombi Kotto it was highest within children of school age. Mean water contact risk scores were higher among infected participants (*P* < 0.001) and also higher among participants in Barombi Kotto compared to Barombi Mbo (*P* < 0.001), Table 2. Use of lake water for domestic purposes was associated with infection (χ^2^(1) 6.84, *P* = 0.009). Risk factors associated with egg-patent infection are shown in Table 3, the strongest associations being lake location and sourcing water from it.

**Table 2:** Mean risk score calculated from questionnaires (study participants with and without egg-patent urogenital schistosomiasis)

		Mean Risk Score (All) (SD) [Range]	Significance	Mean Risk Score (Infected Cases) (SD) [Range]	Significance
All participants		1.73 (1.03) [0.0-4.0]		2.15 (0.89) [0.0-3.5]	
Study site	Barombi Mbo	1.25 (0.90) [0.0-3.5]	<0.001^a^	1.61 (0.89) [0.5-3.0]	0.101^a^
	Barombi Kotto	2.15 (0.97) [0.0-4.0]		2.22 (0.87) [0.0-3.5]	
Age cohort	Adults > 16 years	1.67 (1.07) [0.0-4.0]	0.120^b^	1.89 (0.97) [0.0-3.5]	0.011^b^
	SAC 6 ≤ 16 years	2.03 (0.89) [0.0-3.5]		2.59 (0.57) [1.0-3.3]	
	PSAC ≤6 years	1.18 (0.90) [0.0-3.5]		2.00 (0.87) [1.0-3.5]	

^a^ Mann–Whitney U-test; ^b^ One-way ANOVA. Mean risk score assigned based on responses to questions on swimming, washing, fishing and other behaviours (laundry or water crossing by participants), assigned an arbitrary weighting according to frequency of exposure (0 for never; 0.25 for monthly; 0.5 for weekly; 1.0 for daily)

**Table 3:** Bivariate logistic regression analysis of location, demography and behaviour and egg-patent urogenital schistosomiasis

Variable	Outcome category	Unadjusted OR	95% CI	*P* value
Location	Barombi Mbo Barombi Kotto	- 7.76	3.85-15.66	<0.001
Gender	Male Female	- 1.25	0.62-2.56	0.531
Age cohort	PSAC ≤6 years SAC 6 ≤ 16 years Adults > 16 years	- 2.52 1.57	1.25-5.05 0.79-3.11	0.009 0.196
Previous treatment	≥1 in last five years None in last five years	- 1.48	0.88-2.49	0.114
Swimming	Never swims in the lake Swims in the lake	- 1.64	0.98-2.76	0.062
Washing	Never washes in the lake Washes in the lake	- 1.56	0.95-2.60	0.077
Fishing	Never goes fishing in the lake Goes fishing in the lake	- 2.05	1.09-3.85	0.026
Water sourcing	Does not use lake water Uses lake water	- 3.37	1.40-8.08	0.007

**Fig. 2:**
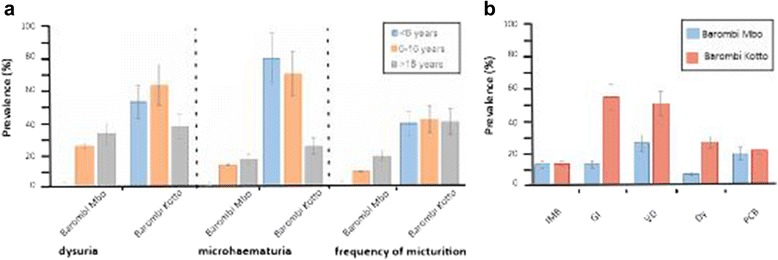
Bar charts of prevalence of sign and symptoms reported by participant or by diagnostic testing. **a** Several urological parameters differ between Barombi Mbo and Barombi Kotto. **b** Key symptoms of female genital schistosomiasis differ between crater lake populations

Urinary symptomatologies were more frequently reported at Barombi Kotto, with school children reporting the highest frequencies of dysuria (χ^2^(2) 11.076, *P* = 0.004) and macrohaematuria (χ^2^(2) 11.076, *P* = 0.004, Fig. 2a). Proteinuria, abnormal urology and albuminuria were all associated with infection and showed positive correlations with egg counts (data not shown). In the subgroup of females ≥16 years (*n* = 67), 54 (81%) completed the FGS questionnaire, with 27 women (50%) reporting ≥1 symptom. Presence of any four FGS symptoms was correlated with infection (*P* = 0.023). FGS symptoms were more commonly reported in Barombi Kotto (Fig. 2b).

### Prevalence and intensity of STH

According to faecal sampling, overall prevalence of STH in 2016 was 6.3% (*n* infected = 15; 95% CI 3.6–10.0, including 2 cases of *T. trichiura*-hookworm co-infection). From Baermann and charcoal culture methods, no larva of *St. stercoralis* was encountered, although hookworm larvae were recovered from two charcoal cultures, and no egg of *A. lumbricoides* was observed. Eggs of *T. trichiura* were observed in Kato-Katz smears prepared at Barombi Mbo with a general prevalence of 8.0%, Table 4. Hookworm prevalence as measured by Kato-Katz sampling varied between 2.0–4.0% between the crater lakes. In comparison to 2004 data, there have been substantial reductions in STH prevalence at both Barombi Mbo and Barombi Kotto, with a relative decrease of more than 79.0%, Table 4.

**Table 4:** Prevalence of each soil-transmitted helminth by location data collected in 2004^a^ and 2016

	Barombi Mbo		Barombi Kotto island		Barombi Kotto mainland	
	2004	2016	2004	2016	2004	2016
	*N* (%) [95% CI]	*N* (%) [95% CI]	*N* (%) [95% CI]	*N* (%) [95% CI]	*N* (%)[95% CI]	*N* (%) [95% CI]
Roundworm	21/30 70.0 (51.3-85.6)	0/113 0.0 (0.0-3.2)	10/57 17.5 (8.7-30)	0/112 0.0 (0.0-3.2)	8/58 13.8 (6.1-25.3)	0/29 0.0 (0.0-12.0)
Whipworm	23/30 76.7 (58.4-90.8)	9/113 8.0 (3.7-15.0)	20/57 35.1 (23.4-49.6)	0/112 0.0 (0.0-3.2)	26/58 44.8 (3.82-59.7)	0/29 0.0 (0.0-12.0)
Hookworm	9/30 30.0 (15.2-49.3)	4/113 3.5 (0.9-8.8)	7/57 12.3 (5.1-24.4)	3/114 2.6 (0.5-7.5)	15/58 25.9 (15.3-39.7)	1/29 3.4 (0.1-18.0)

^a^from Tchuenté 2004

### WASH-related findings

**Fig. 3:**
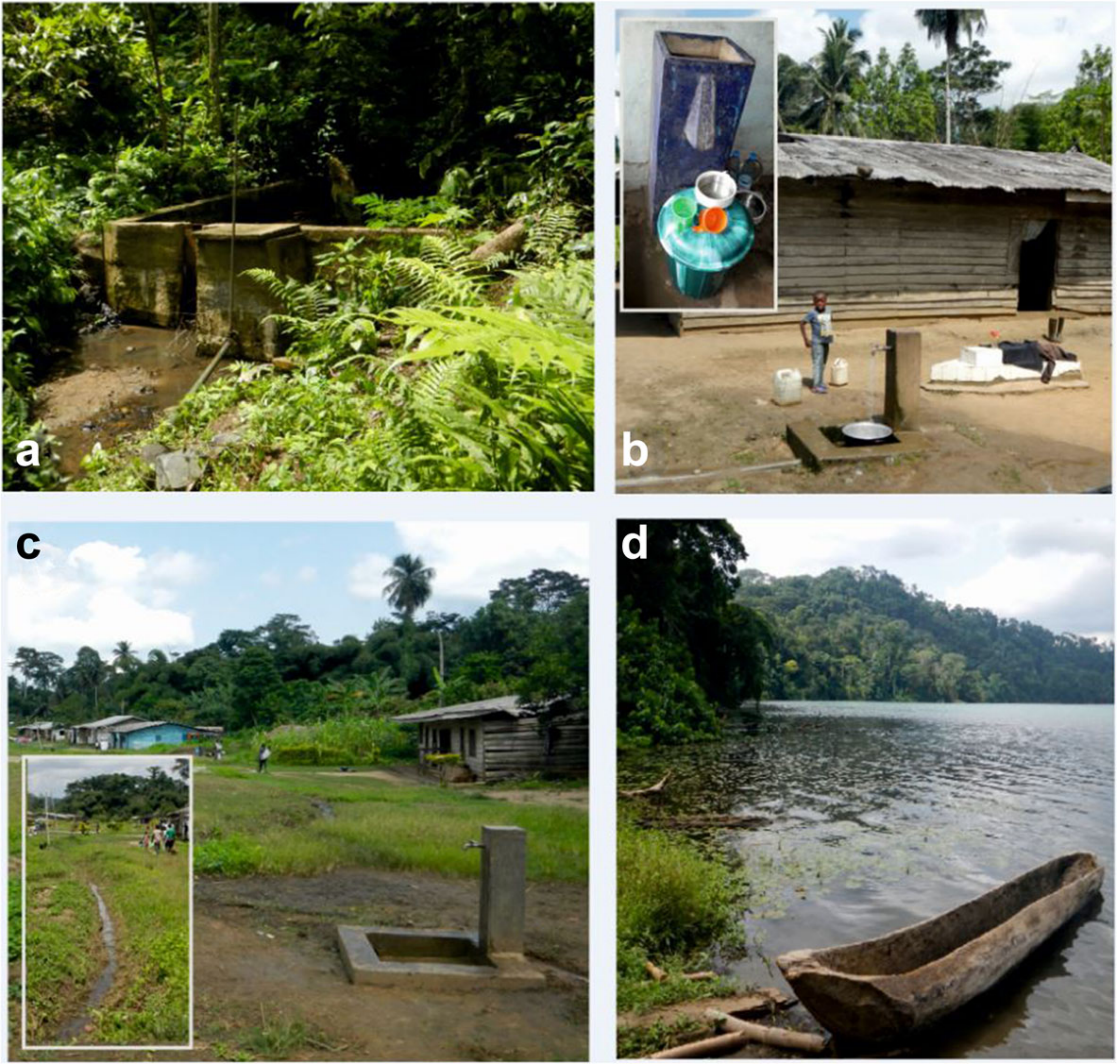
Photographs of pertinent WASH intrastructures found at Barombi Mbo. **a** Gravity flow reservoir located 2 km away from the village. **b** Typical housing of Barombi Mbo with piped water stand nearby houshold grave [inset. Sandbox water filter for household drinking water]. **c** Village stand pipe for water; note open field drainage of water [inset. Several open drains from piped water coalesce in the center of the village and form an open drain, as yet not colonised by snails which are found downstream: *B. forskalii* are found at site A5 (see Fig. 4c)]. **d** Lakeshore canoe landing site where many *B. truncatus* were found often in discarded cut bamboo and plastic containers amongst the water lilies and aquatic vegetation

WASH-related infrastructure and associated hygiene behaviours differed between lakes. In 2008, a fully functioning gravity flow, closed piped water system with sand box filter, had been installed at Barombi Mbo by a USA-based non-government development organisation which had also provided individual households with sand-filter boxes within households, and many of these were in use, *see* Fig. 3. Eighty-three per cent of participants at Barombi Mbo reported use of improved drinking water whereas all Barombi Kotto participants reported using unimproved drinking water which was taken from a nearby waterfall (use of lake water χ^2^(1) 10.891, *P* = 0.001). However, use of improved sanitation facilities was similar for each village; being 35% of participants at Barombi Mbo and 39% of participants at Barombi Kotto (*P* = 0.622). In terms of behaviour there was no significant difference between villages for reported shoe wearing (55% of Barombi Mbo residents, and 50% of Barombi Kotto residents, always wearing shoes; *P* = 0.540), and mild differences for reported soap use when handwashing (28% for Barombi Mbo and 38% for Barombi Kotto always using soap; *P* = 0.030).

### Treatment history with anthelminthics

Significantly more residents of Barombi Kotto (73.2%) reported having received anthelminthic treatment previously, compared to residents of Barombi Mbo (47.9%, *P* = <0.001). Participants who reported a history of previous infection were associated with having current schistosome egg-positive urine (χ^2^(1) 13.25, *P* < 0.001), as were those who reported never having received treatment (χ^2^(1) 4.04, *P* = 0.045), with positive associated risk factors, Table 3. Reported treatment coverage was lowest in the PSAC group.

### Malacology surveys

**Fig. 4:**
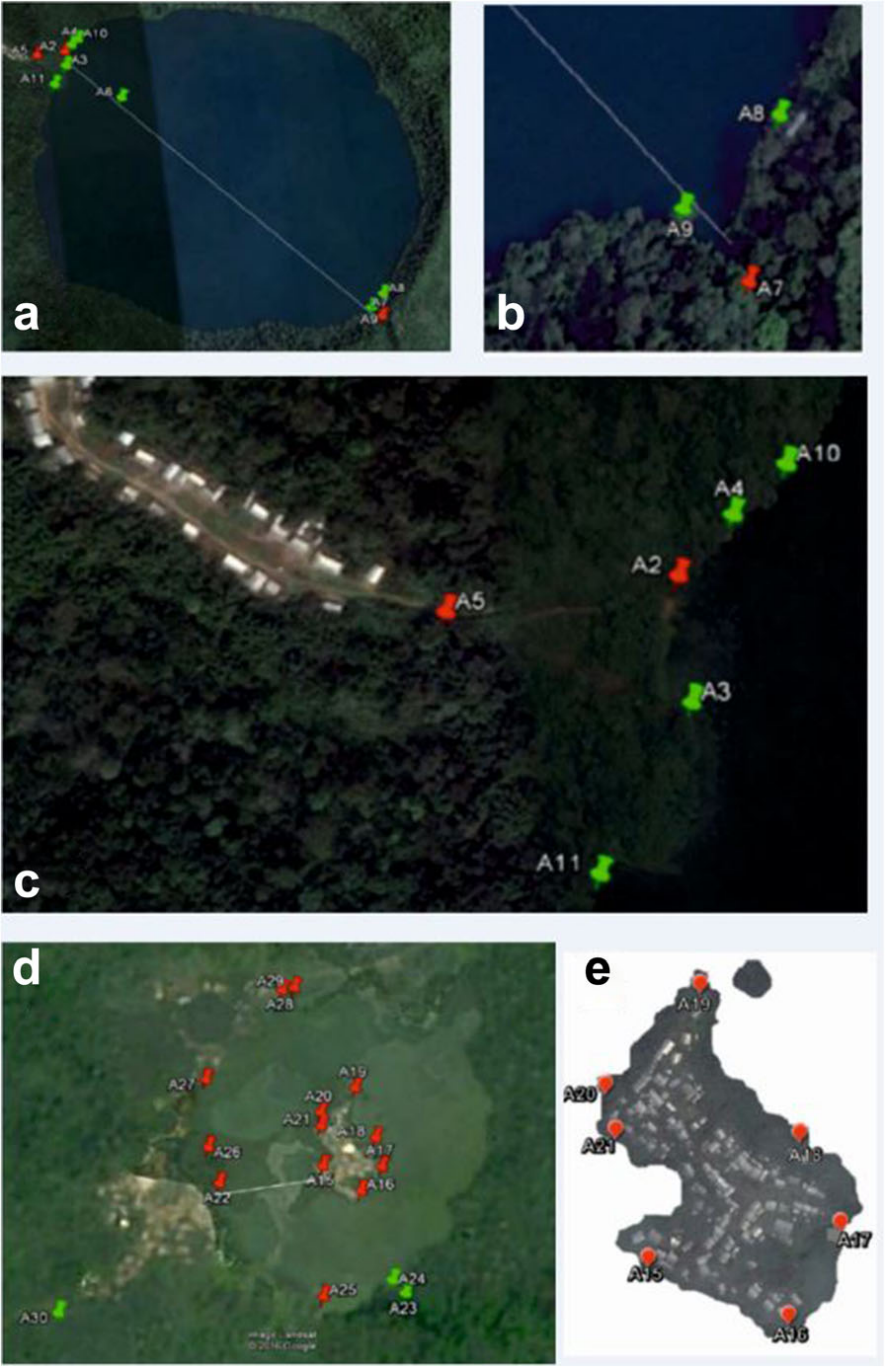
Schematic plot of snail collecting sites at Barombi Mbo and Barombi Kotto. **a** Barombi Mbo is aprroximately 2.5 km in diameter with human water contact sites at either side of the lake, **b** southern lake shore, **c** northern lake shore, all sample sites (green pins); sites where snails were found are indicated by red pins. **d** Barombi Kotto is approximately 1.2 km in diameter with water contact sites largely spread around the whole shoreline. Location of all sampling sites (green pins); sites where snails were found are indicated by red pins. **e** Location of sampling site on the central inhabited island of Barombi Kotto

Thirteen collecting sites around Barombi Mbo (A2 - A14), 7 around the central island of Barombi Kotto (A15 - A21) and 9 sites around Barombi Kotto lake perimeter (A22 – A30; note A30 was an outflow stream) were surveyed, see Fig. 4. Water chemistry readings were collected at each site and for Barombi Mbo the average temperature was 30.2 °C, mean pH of 7.9 and mean conductivity was 64.3 μS, for Barombi Kotto, the average temperature was 31.2 °C, mean pH of 9.6 and mean conductivity was 202.1 μS. The waters of Barombi Mbo have significantly lower pH and conductivity than Barombi Kotto (all *P* < 0.0001). Fig. 4 depicts the survey locations at Barombi Mbo; a total of 166 *Bulinus* were found (*B. truncatus* (*n* = 89) and *B. forskalii/camerunensis* (*n* = 77)) occurring at three sites (A2, A5 and A7). Since *B. forskalii* and *B. camerunensis* are only distinguishable upon molecular analysis, those snails that were collected but were not subjected to DNA analysis were referred to as *B. forskalii/camerunensis*. No snail from Barombi Mbo was observed to shed schistosome cercariae.

**Fig. 5:**
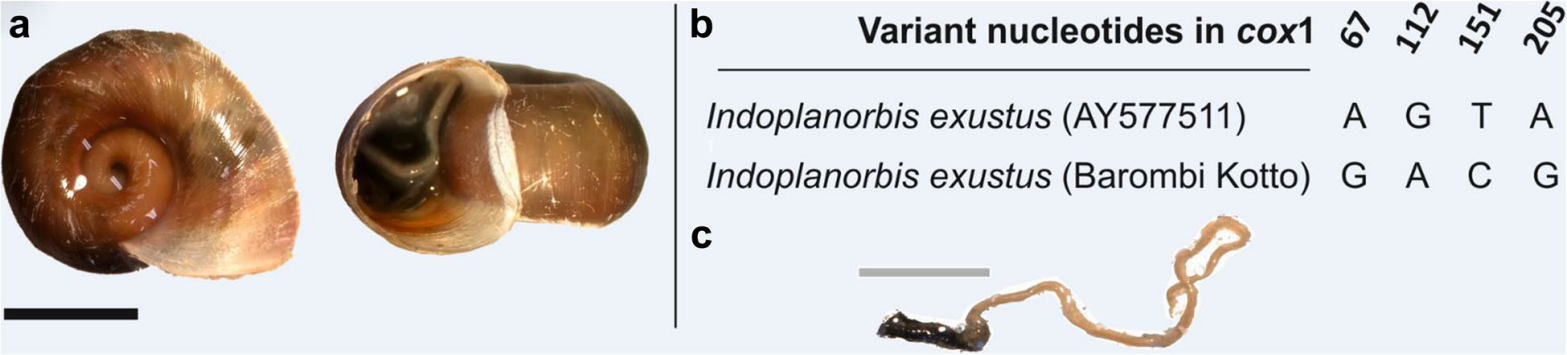
*Indoplanorbis exustus* from Barombi Kotto. **a** Shell photographs (scale bar 0.5 cm). **b** Variant nucleotides within the *cox*1 upon comparison with reference DNA sequence. **c** Typical male genitalia from dissected snails having a characteristic short preputium (without the accessory gland characteristic of *Helisoma* spp.) and long penis sheath which clearly resembles the anatomy of *Bulinus*

At Barombi Kotto apart from at three sites, snails were present in all sampling locations where twice as many snails per site were found along the island shoreline versus the lake perimeter. In total, 285 *Bulinus forskalii/camerunensis* were found and were of mixed sizes indicating all levels of snail maturity. An unexpected discoidal planorbid, tentatively identified upon shell morphology as either (?)*Helisoma* or (?)*Indoplanorbis*, was very common, with a total of 220 snails found. Following an anatomical dissection and inspection of the male genitalia together with an analysis of the *cox*I (see Fig. 5), this species was identified as *Indoplanorbis exustus* and is a first report of this Asian invasive species in Cameroon. Two *B. camerunensis* snails were observed to be shedding schistosome cercariae (site A20), and strigeid and gymnocephalous cercariae were encountered at sites A2 and A26.

### Molecular characterisation of snails and schistosomes

Real-time PCR assays detected *Schistosoma* DNA in three *B. truncatus*, all from site A2, at Barombi Mbo. Partial *cox*I sequences were obtained for three *B. truncatus* and three *B. forskalii/camerunensis,* and were used in a BLAST search of GenBank. Exact matches were found with *B. truncatus* (GenBank: KJ157409.1) [58] and *B. camerunensis* (GenBank: AM286309.2) [54]. At Barombi Kotto, real-time PCR analyses detected the presence of *Schistosoma* DNA in seven *B. forksalii/camerunensis* and in two *Indoplanorbis exustus* (all from site A20). Partial *cox*I sequences were obtained from six *B. forskalii/camerunensis* and three *Indoplanorbis exustus* finding exact matches upon BLAST searches with *B. camerunensis* (GenBank: AM286309.2) [54] and very high similarity (98.8%) with *I. exustus* (GenBank: AY577511.1) [59].

The partial *cox*1 and ribosomal ITS sequences were successfully generated from 11 schistosome miracidia from Whatman® FTA® cards. A BLAST search of GenBank revealed exact matches with *cox*1 haplotype *S. haematobium* H1 found previously from material collected from Barombi Mbo (GenBank: JQ397366) and Barombi Kotto (GenBank:JQ397367) and ITS *S. haematobium* sequence (GenBank:JQ595394).

## Discussion

The fauna of these crater lakes of Cameroon [27, 28, 60–63] have been the subject of much study as well as their surrounding geomorphology [64] and sedimentology [65], which provides strong evidence of human settlement some 2 800 years ago [66]. Today, each lake has several unique features, either natural or of manmade-origin. Barombi Mbo is much larger (2 500 m *v.* 1 200 m in diameter), substantially deeper (111 m *v.* 6 m), does not have a central inhabited island and is more oligotrophic (i.e. poor in nutrients) than Barombi Kotto. The majority of the shoreline of Barombi Mbo is very steeply shelved and largely not accessible by foot. In terms of limnology, while surface water temperatures are very similar, the pH and water conductivity are clearly different. Barombi Kotto is more alkaline with higher conductivity with more turbid, typically green water supporting copious phytoplankton [62].

Several of these differences were noted over 40 years ago reporting pH (6.3-7.0 v. >9.0) and conductivity (39.0 μS *v.* 150 μS) [35, 61, 62]. It is interesting that current water conductivity readings seem to have increased in both lakes. This could be due to anthropogenic influences such as pollution or by progressive eutrophication from surrounding cocoa plantations, the main cash crop. In comparison to other water bodies, the freshwater snail fauna here is meagre, with prosobranch species absent [50] and little planorbid biodiversity [67], for only 3 species of *Bulinus*, and an invasive Asian species *Indoplanorbis exustus* [68], were present. This latter species is an important intermediate host of Asiatic schistosomes and several food borne trematodes [69]. It has dispersed across continents, likely spread by man through the aquatic plant trade or by pisciculture through restocking [70]. Experimental infections of *I. exustus* with African schistosomes have not been able to demonstrate compatibility as yet [68], so this species may be playing a beneficial role as a biological control agent by drawing away schistosome miracidia that might otherwise develop in local *Bulinus*. This is supported by the detection of *Schistosoma* DNA in snails at site A20 but further study of the experimental compatibility of this species with local schistosomes would be advisable, as well as, increasing vigilance for other trematode diseases it may be able to transmit.

In terms of schistosomiasis and STH, the importance of the former was first described in these crater lakes in 1953 [36], with serious attempts to control its impact some twenty years later. These involved a combination of focal mollusciciding with frescon and mass treatment of patients with either ambilhar or niridazole [32–35]. While there was much promise in these efforts, they were not sustained, for each control tool became obsolete, and similar integrated approaches were largely abandoned [7, 71]. There were, however, attempts to use alternative molluscicides (i.e. bayluscide) in other parts of Cameroon [6, 31, 72], but only after the anthelminthic praziquantel became available and more affordable [73] could large-scale interventions against schistosomiasis become scalable [74]. This was further enhanced by the donation by Merck-KGaA (http://unitingtocombatntds.org/endorsement/merck-kgaa), so delivery of praziquantel could be integrated with either mebendazole or albendazole, expanding the remit of preventive chemotherapy campaigns to tackle several diseases simultaneously and, more importantly, at increasing scales of operation given the endemic zone of schistosomiasis and STH in Cameroon [9–11, 13, 19, 38, 75–77].

Nonetheless schistosomiasis and STH within these crater lakes have continued to cause morbidity for decades [15, 44], although the reported declines in prevalence of STH from 2004 are particularly impressive, and the absence of *Strongyloides* spp. has been confirmed. Even today, however, the disease burden of urogenital schistosomiasis is not fully appreciated, particularly in reference to FGS [78] or in its burden in preschool-aged children [79]. This was each clearly demonstrated in Barombi Kotto by raised signs and symptoms of FGS, for example dyspareunia, in women and that a quarter of PSAC had egg-patent infections. It is therefore appropriate to consider better targeting of preventive chemotherapy to these vulnerable populations if ensuing morbidity is to be better averted. This should become a subject of further investigation as interventions are targeted towards addressing these obvious gaps.

In the context of reported anthelminthic treatment coverage of 73.2% and 47.9%, ascertained in SAC and adults, at Barombi Kotto and Barombi Mbo respectively, there is still some room for improvement. Options for more intensified control interventions could include increasing frequency of treatment by offering selective treatment to those who request it throughout the year, alongside current MDA interventions which could operate at treatment cycles shorter than the current annual provision. In terms of gaining and sustaining control at Barombi Kotto, biannual MDA treatment could accelerate better towards expected declines in parasitaemia by mitigating reinfection more strongly. Previously the reinfection dynamics of schistosomiasis have been shown to be variable at mixed infection foci [19], but given that autochthonous transmission of intestinal schistosomiasis is not possible here as *Biomphalaria* is absent, future studies could shed more light on re-infection patterns. This should specifically address each of the currently infected demographic groups to help tailor future retreatment cycles.

**Fig. 6:**
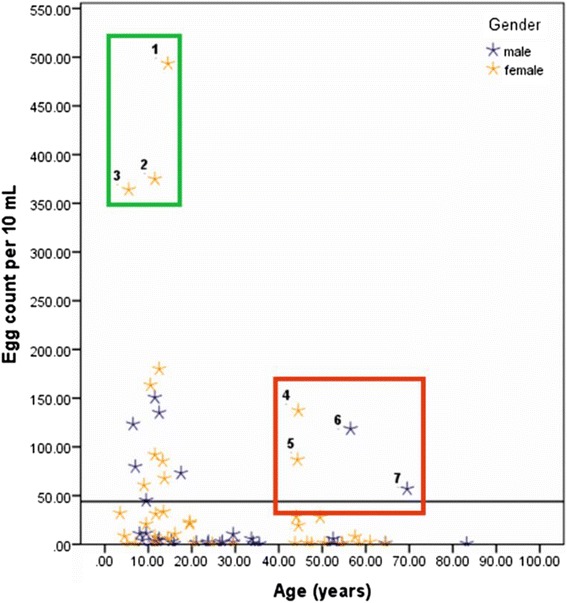
Scattergram of intensity of egg-patent infection with participant age reveals that there are individuals within each demographic group that have much higher than average egg counts (individuals 1–3 being school-aged children and individuals 4–7 being adults). It is likely that these individuals are most responsible for transmission of the disease, therefore future targetting of treatment to these individuals specifically would be worthwhile

An important additional point to consider when planning intensified control interventions is deciding when and how MDA campaigns should transition to test and treat strategies and in which demographic groups. The need to do so is exemplified by Fig. 6, where two SAC and one PSAC (see Fig. 6, numbered 1–3 inside the green box) from Barombi Kotto reported to wash regularly in the lake and complained of dysuria; furthermore four adults at Barombi Kotto (two males and two females) were identified as having egg counts far above the mean (see Fig. 6, numbered 4–7). Three reported to have had urogenital previously and had received treatment previously although all swam and washed in the lake, and both males fished. These findings support typical profiles of infected individuals, for example, high risk water contact, evidence of urological morbidity and sporadic treatment history. Some future targeting is needed to better identify these individuals such that all are treated more regularly to ensure that their inputs to environmental contamination are mitigated as best possible.

As preventive chemotherapy does not guard against reinfection, broadening the scope of intensified interventions against schistosomiasis and STH to go beyond that of current anthelminthic treatment regimens alone, is crucial; hence WASH-related interventions that reduce environmental transmission and exposure are important [14, 17]. Most significantly, since 2008 the inhabitants of Barombi Mbo have benefited from the provision of an extensive improved water supply, bringing piped water close to all households, Fig. 3. It would seem likely that in addition to preventive chemotherapy this WASH intervention has clearly helped reduce those diseases where faecal-oral transmission occurs, given that household sanitation was relatively similar between locations. Whilst the transmission of schistosomiasis shares several features with STH, urogenital schistosomiasis sets itself apart by being transmitted within urine and not within stool.

It is open to conjecture what impact an improved water supply might have at Barombi Kotto in terms of transmission of urogenital schistosomiasis, if similarly installed and used since risk factors at each lake are clearly different, Table 2. While there are direct and indirect impacts of improving WASH for those who would no longer depend on drawing domestic water from the lake but, however, for those that fish, cross by canoe or play in the lake their cumulative water contact levels, and risk of infection, might not significantly reduce. All of the latter are significant local risk factors, see Table 3. Future recourse to snail control with focal molluscicides could be beneficial, and particularly insightful when set against previous attempts locally [32–34]. Historically, however, the prevalence of STH was much less than that at Barombi Mbo and in explanation, the volcanic soil type at Barombi Kotto might not be favourable for STH. Nevertheless, a safer water supply for the inhabitants on the Barombi Kotto island would be beneficial as a significant complementary factor for intensified treatment interventions and for several other reasons outside that of current STH and schistosomiasis control perspectives.

## Conclusions

Our findings have implications in better tailoring and evaluating future interventions against schistosomiasis and STH, as several important lake-specific heterogeneities have been revealed. STH is currently at very low levels while urogenital schistosomiasis is of greatest concern at Barombi Kotto. The study of the epidemiological dynamics of each disease in these crater lakes provides a context for future assessment of intensified control, for example, it is most insightful for urogenital schistosomiasis in terms of gaining and sustaining control at Barombi Kotto or in moving towards local interruption of transmission at Barombi Mbo.

## Additional file

Additional file 1: Multilingual abstracts in the five official working languages of the United Nations. (PDF 906 kb)

## Abbreviations

CCA: Circulating cathodic antigen
DNA: Deoxyribonucleic acid
FGS: Female genital schistosomiasis
GPS: Global positioning system
ITS: Internal transcribed spacer
JMP: WHO/UNICEF joint monitoring programme for water supply and sanitation
MDA: Mass drug adminsitration
NTDs: Neglected tropical diseases
PCR: Polymerase chain reaction
PSAC: Pre-school-aged children
SAC: School-aged children
STH: Soil transmitted helminthiasis
WASH: Water, sanitation and hygiene
