# Supplementary material for: Urogenital schistosomiasis and soil-transmitted helminthiasis (STH) in Cameroon: An epidemiological update at Barombi Mbo and Barombi Kotto crater lakes assessing prospects for intensified control interventions
Source: Infect Dis Poverty. 2017 Feb 27;6:49. doi: 10.1186/s40249-017-0264-8 (PMC5327519; doi:10.1186/s40249-017-0264-8)
Supplement: Additional file 1: — Multilingual abstracts in the five official working languages of the United Nations. (PDF 906 kb) [file 40249_2017_264_MOESM1_ESM.pdf]

Translation of the abstract into the five official working languages of the United Nations

البلهارسيا البولية التناسلية والديدان الطفيلية المنقولة عن طريق التربة (STH) في الكاميرون: تحديث وبائية في بحيرات بارومبي مبو وبارومبي كوتو البركانية لتقييم دلائل للمكافحة المكثفة

سوزي جي. كامبل، جي. راسل ستوثيرد، فاي أوهالرون، ديبورا سانكي، تيموثي ديورانت، ديودوني إلوندي أومبيد، جولاديس جومكام تشونتي، بوني إل. وبستر، لوكاس كانينجهام، إي. جيمس لا كورس، لويس ألبرت تشوإم تشانتى

الملخص

الخلفية: تشتهر بحيرات **بارومبي مبو وبارومبي كوتو** البركانية بأنها بؤر انتقال للبلهارسيا والديدان الطفيلية المنقولة عن طريق التربة وذلك بعد إجراء عدة مبادرات هامة للمكافحة في السابق. لجمع المعلومات الوبائية المعاصرة، تم إجراء مسح مقطعي يشمل: علامات وأعراض المرض، وتاريخ العلاج الفردي، المياه المحلية والصرف الصحي والعوامل ذات الصلة بالنظافة الصحية (WASH) ومراقبة الرخويات، مع التوصيف الجزيئي للعينات.

الطرق: في كل بحيرة، تم إجراء مسح مجتمعي مستعرض وذلك باستخدام التصنيف الطفيلي لمزيج من البراز والبول ، وإجراء مقابلة مع نماذج استبيانات. وقد شارك إجمالي 338 من الأطفال والبالغين. وتميزت المواد من أنواع القواقع والطفيليات بأساليب الحمض النووي.

النتائج: كان الانتشار واضحاً لبويضات البلهارسيا البولية التناسلية 8.7٪ في **بارومبي مبو** (جميع الإصابات خفيفة) و40.1٪ في **بارومبي كوتو** (عدوى شديدة 21.2٪). ولا توجد بلهارسيا معوية. في **بارومبي كوتو،** ذكر كثير من النساء بصورة ملحوظة العلامات والأعراض المصاحبة لمرض بلهارسيا الأعضاء التناسلية للإناث. وبينما كان هناك تحسن واسع في الآونة الأخيرة في البنية التحتية ذات الصلة بالمياه والصرف الصحي، في **بارومبي مبو،** كانت دلائل خطر التعامل مع الماء أعلى بين المشاركين المصابين بالبلهارسيا (*p*<0.001) وفي **بارومبي كوتو** عامة (*p*<0.001). عبر كل من البحيرتين، كان متوسط انتشار الديدان الطفيلية المنقولة عن طريق التربة STH منخفضاً جداً (6.3٪) وهذا يدل على وجود انخفاض هائل من 79.0٪ خلال العقد الماضي، ولم يعثر على الأسطوانية البرازية *Strongyloides stercoralis*n أو ديدان الاسكارس (*Ascaris lumbricoides*). تم فحص إجمالي 29 موقعاً لأخذ عينات من المياه العذبة للقواقع، و 13 في **بارومبي مبو** و 16 في **بارومبي كوتو**. اختلفت كيمياء المياه بشكل ملحوظ (p<0.0001) بين البحيرتين لكل من متوسط الرقم الهيدروجيني (7.9 مقابل 9.6) ومتوسط التوصيل الكهربائي (64.3 ميكرو ثانية مقابل 202.1 ميكرو ثانية) على التوالي. لوحظ وجود اثنان فقط من قواقع Bulinus camerunesis على الجزيرة الوسطى **لبارومبي كوتو** تطرحان البلهارسيا schistosome cercariae، ولكن تم الكشف عن الحمض النووي للبلهارسيا في وقت لاحق في عينات *Bulinus* من كلا البحيرتين وكذلك في المقوقعات الهندية *Indoplanorbis exustus* ، وهي نوع اجتياحي من آسيا.

الاستنتاجات: مستويات الديدان الطفيلية المنقولة عن طريق التربة STH حالياً منخفضة للغاية في حين أن البلهارسيا البولية التناسلية هي الأكثر إثارة للقلق في **بارومبي كوتو**. يبرز هذا التقييم فرصة فريدة لمزيد من الدراسة للديناميكيات الوبائية في هذه البحيرات البركانية ، ولتقييم مستقبل التدخلات المكثفة سواء من حيث اكتساب والحفاظ على السيطرة في **بارومبي كوتو** أو في المضي قدماً نحو الإيقاف الموضعي لانتقال كلا المرضين في **بارومبي مبو**.

Translated from English version into Arabic by Free bird, through

TRANSLATORS WITHOUT BORDERS

喀麦隆尿路血吸虫病和土源性蠕虫病：Barombi Mbo 和 Barombi Kotto 两个火山口湖地区流行现状及强化干预后的效果评价

Suzy J. Campbell, J. Russell Stothard, Faye O’Halloran, Deborah Sankey, Timothy Durant, Dieudonné Eloundou Ombede, Gwladys Djomkam Chuinteu, Bonnie L. Webster, Lucas Cunningham, E. James La Course, Louis-Albert Tchuem-Tchuenté

摘要

引言：Barombi Mbo 和 Barombi Kotto 两个火山口湖是著名的血吸虫病和土源性蠕虫病传播地区，并且之前开展了几项重要的控制项目。为收集最新的流行病学情况，开展了一项横断面调查，内容包括疾病症状和体征、个体治疗情况，当地水、厕所和个人卫生情况（WASH）相关指标及螺类监测情况，标本采用分子生物学方法鉴定。
方法：在两个湖区，同时收集粪便和尿液标本进行检测，并且采用预先设计的问卷进行问卷调查。共有 338 名儿童和成人参加。螺类和寄生虫标本通过 DNA 方法鉴定。
结果：Barombi Mbo 湖区尿路血吸虫病的虫卵阳性率为 8.7%，且均为轻度感染；而 Barombi Kotto 湖区则为 40.1%，且 21.2%为重度感染；未发现肠道血吸虫病（曼氏血吸虫病）。Barombi Kotto 湖区，更多女性报告有生殖道血吸虫病相关体征和症状。而 Barombi Mbo 湖区 WASH 指标近来有显著改善，血吸虫感染者的疫水接触指标显著高于非感染者（$P<0.001$），Barombi Kotto 湖区显著高于 Barombi Mbo 湖区 （$P<0.001$）。在两个湖区，土源性蠕虫病患病率较低，平均为 6.3%，表明过去十年感染率下降了 79.0%。未发现粪类圆线虫（*Strongyloides stercoralis*）和蛔虫（*Ascaris lumbricoides*）。共调查了 29 处淡水点进行螺类检测，其中 Barombi Mbo 湖区 13 处， Barombi Kotto 湖区 16 处。两个湖区水化学指标（pH 均值和导电性均值）均有显著性差异（$P<0.0001$），pH 均值分别为 7.9 和 9.6，导电性均值分别为 64.3 µS 和 202.1 µS。仅在 Barombi Kotto 湖区中心岛屿上发现了 2 只 *Bulinus camerunesis* 溢出血吸虫尾蚴，但是两个湖区采集的 *Bulinus* 和来自亚洲的入侵生物 *Indoplanorbis exustus* 样本中均检出血吸虫 DNA 样本。
结论：两个湖区土源性蠕虫病目前处于较低流行水平，而尿路血吸虫病在 Barombi Kotto 更严重。该评估调查强调了在这两个火山口湖地区继续开展流行病学动态调查的重要性，以进一步评估干预措施的效果，包括 2 种疾病在 Barombi Kotto 湖区的可持续性控制成效和在 Barombi Mbo 湖区朝着传播阻断目标迈进的情况。

Translated from English version into Chinese by Men-Bao Qian, edited by Yang Pin, through

CHINESE CENTER FOR DISEASE CONTROL AND PREVENTION
NATIONAL INSTITUTE OF PARASITIC DISEASES

Schistosomiase urogénitale et helminthiase transmise par le sol (HTS) au Cameroun : actualisation épidémiologique dans les lacs de cratère de Barombi Mbo et Barombi Kotto en vue d'évaluer les perspectives d'interventions de lutte intensifiées

Suzy J. Campbell, J. Russell Stothard, Faye O'Halloran, Deborah Sankey, Timothy Durant, Dieudonné Eloundou Ombede, Gwladys Djomkam Chuinteu, Bonnie L. Webster, Lucas Cunningham, E. James La Course, Louis-Albert Tchuem-Tchuenté

Résumé

Contexte: Les lacs de cratère de Barombi Mbo et Barombi Kotto sont des foyers bien connus de transmission de la schistosomiase et de l'helminthiase transmise par le sol, qui ont déjà fait l'objet de plusieurs initiatives de lutte importantes. Afin de recueillir des informations épidémiologiques à jour, une étude transversale des signes et symptômes de maladie, des historiques individuels de traitement, des facteurs locaux relatifs à l'eau, à l'assainissement et à l'hygiène (EAH) et une surveillance malacologique avec caractérisation moléculaire des spécimens ont été réalisées.
Méthodes: Nous avons réalisé autour de chaque lac une étude transversale des communautés combinant des de prélèvements parasitologiques de selles et d'urines et des entretiens avec des formulaires pro forma. Au total, 338 enfants

et adultes ont participé à l'étude. Le matériel issu d'espèces d'escargots et de parasites ont été caractérisés par analyse de l'ADN.

Résultats: La prévalence révélée par les œufs de la schistosomiase urogénitale était de 8,7 % au Barombi Mbo (infestations peu intenses dans tous les cas) et 40,1 % au Barombi Kotto (21,2 % d'infestations intenses). Il n'y avait pas de schistosomiase intestinale. Autour du lac Barombi Kotto, les femmes rapportant des signes et symptômes associés à la schistosomiase génitale étaient significativement plus nombreuses. Bien que l'infrastructure EAH se soit considérablement améliorée depuis peu autour du Barombi Mbo, les scores de risque par contact avec l'eau étaient plus élevés parmi les participants infestés par la schistosomiase ($P < 0,001$), ainsi qu'autour du Barombi Kotto en général ($P < 0,001$). La prévalence moyenne des HTS était très faible (6,3 %) autour des deux lacs et a connu une diminution impressionnante (79,0 %) sur les dix dernières années. Nous n'avons trouvé ni *Strongyloides stercoralis* ni *Ascaris lumbricoides*. Au total, 29 sites de prélèvement d'eau douce ont été inspectés à la recherche d'escargots : 13 dans le Barombi Mbo et 16 dans le Barombi Kotto. La chimie de l'eau était significativement différente ($P < 0,0001$) entre les deux lacs en termes de pH (7,9 contre 9,6 respectivement) et de conductivité moyenne (64,3 µS contre 202,1 µS). Seuls deux spécimens de *Bulinus camerunesis* prélevés sur l'île centrale du Barombi Kotto excrétaient des cercaires de schistosome, mais l'ADN du schistosome a été décelé par la suite chez des *Bulinus* prélevés dans les deux lacs ainsi que chez *Indoplanorbis exustus*, une espèce invasive asiatique.

Conclusions: Les HTS sont actuellement très peu fréquentes et c'est la schistosomiase urogénitale qui constitue le problème le plus préoccupant autour du lac Barombi Kotto. Cette évaluation met en lumière une occasion unique d'explorer davantage la dynamique épidémiologique de ces lacs de cratère afin d'évaluer de futures interventions intensifiées aussi bien pour faire durablement reculer l'infestation autour du Barombi Kotto que pour obtenir l'interruption locale de la transmission des deux maladies autour du Barombi Mbo.

Translated from English version into French by Suzanne Assenat, through

TRANSLATORS WITHOUT BORDERS

Урогенитального шистосомоз и передаваемые через почву гельминтозы (СТГ) в Камерун: эпидемиологическое обновление на Баромби Мбо и Баромби Котто кратер озера оценки перспективы для активизации мероприятий по усилили контроль

Suzy J. Campbell, J. Russell Stothard, Faye O'Halloran, Deborah Sankey, Timothy Durant, Dieudonné Eloundou Ombede, Gwladys Djomkam Chuinteu, Bonnie L. Webster, Lucas Cunningham, E. James La Course, Louis-Albert Tchuem-Tchuenté

Фон: кратера озера Баромби Мбо и Баромби Котто хорошо известны передача очагами шистосомоза и передаваемых через почву гельминтоза имея ряд важных инициатив по контролю над ранее. Для сбора современной эпидемиологических информация, исследование поперечного сечения было проведено с учетом: признаки и симптомы болезни, индивидуальные истории лечения, местная вода, санитария и гигиена (ВСГ) - связанные факторы и малакологический наблюдения, с молекулярной характеристики образцы.

Методы: На каждом озере, исследование поперечного сечения сообщество было проведено с использованием сочетание стула и отбора проб мочи паразитологических, а также интервью с проформы опросные листы. В

общей сложности 338 детей и взрослых участвовали. Материал из улиток и паразитные виды были охарактеризованы методами ДНК.
Результаты: Яйцо-патент распространенность урогенитального шистосомоза составила 8,7% при Баромби Мбо (все интенсивности света инфекции) и 40,1% в Баромби Котто (21,2% тяжелой интенсивности инфекции). Кишечный шистосомоз отсутствовал. В Баромби Котто, значительно больше женщины сообщили признаки и симптомы, связанные с женских половых шистосомоз. В то время как было обширное недавнее улучшение в ВСГ, связанных с инфраструктурой в Баромби Мбо, оценки риска воды контакта были выше среди шистосоматоз-инфицированных участников (*P* <0,001) и на Баромби Котто в целом (*P* <0,001). Через обоих озерах, в виду распространенность СТГ была очень низкой (6,3%), что свидетельствует впечатляющее снижение 79,0% за последнее десятилетие; ни Стронгилоидез стеркоралис, ни человеческая аскарида были найдены. В общей сложности 29 участков отбора проб пресной воды были проверены на улиток, 13 в Баромби Мбо и 16 в Баромби Котто; химический состав воды значительно отличались (*P*<0,0001) между озерами как для средней pH (7,9 против 9,6) и средняя проводимость (64,3 мкСм против 202,1 мкСм) соответственно. Только два Булинус камеруненсис найти на центральном острове из Баромби Котто наблюдали пролить шистосомы церкарии, но шистосомы ДНК позже был обнаружен в Булинус выборку из обоих озер, а также в Индопланорбис эксустус, инвазивные виды из Азии.
Выводы: СТГ в настоящее время на очень низком уровне в то время как урогенитальный шистосомоз имеет наибольшее беспокойство у Баромби Котто. Эта оценка подчеркивает уникальную возможность для дальнейшее изучение эпидемиологической динамики в этих озерах кратера, чтобы оценить будущее активизировались мероприятия как с точки зрения получения и поддержания контроля на Баромби Котто или движется в направлении локального прерывания передачи обоих заболеваний в Баромби Мбо.

Translated from English version into Russian by Suzy J. Campbell

La esquistosomiasis urogenital y la helmintiasis transmitida por el suelo en Camerún: actualización epidemiológica en los lagos de cráter Barombi Mbo y Barombi Kotto y evaluación de la posible intensificación de las acciones de control

Suzy J. Campbell, J. Russell Stothard, Faye O'Halloran, Deborah Sankey, Timothy Durant, Dieudonné Eloundou Ombede, Gwladys Djomkam Chuinteu, Bonnie L. Webster, Lucas Cunningham, E. James La Course, Louis-Albert Tchuem-Tchuenté

Resumen

Antecedentes: Los lagos de cráter Barombi Mbo y Barombi Kotto son bien conocidos por constituir focos de transmisión de esquistosomiasis y helmintiasis transmitida por el suelo donde en el pasado se han llevado a cabo varias iniciativas de control importantes. Para recopilar información epidemiológica actualizada, se llevó a cabo una encuesta transversal que incluía signos y síntomas de enfermedad, historiales de tratamiento individuales, recursos hídricos locales, factores relacionados con las condiciones sanitarias y la higiene y vigilancia malacológica mediante la caracterización molecular de muestras.
Metodología: En cada lago se realizó un estudio transversal comunitario utilizando una combinación de muestras de heces y de orina y una entrevista mediante cuestionarios predefinidos. Participaron 338 niños/as y adultos/as en total. Se

realizó la caracterización de muestras obtenidas de especies de caracoles y parásitos mediante técnicas de análisis del ADN.

Resultados: La prevalencia de esquistosomiasis urogenital con presencia de huevos fue del 8,7 % en Barombi Mbo (en todos los casos, infecciones leves) y del 40,1 % en Barombi Kotto (el 21,2 %, infecciones graves). No hubo indicios de esquistosomiasis intestinal. En Barombi Kotto, el número de mujeres que refirieron signos y síntomas de esquistosomiasis genital femenina fue considerablemente mayor. A pesar de que recientemente se habían llevado a cabo mejoras considerables en las infraestructuras sanitarias e higiénicas de Barombi Mbo, el riesgo de contacto con el agua era más alto entre los participantes con infecciones por esquistosomiasis ($P<0,001$) y en Barombi Kotto en general ($P<0,001$). La prevalencia media de la helmintiasis transmitida por el suelo entre ambos lagos era muy baja (6,3 %), dando muestras así de una reducción drástica respecto del 79,0 % de la década anterior. No se encontraron *Strongyloides stercoralis* ni *Ascaris lumbricoides*. Se inspeccionaron 29 emplazamientos de muestreo de agua dulce para detectar la presencia de caracoles, 13 en Barombi Mbo y 16 en Barombi Kotto. La composición química del agua difería bastante ($P<0,0001$) entre los lagos, tanto en términos de pH medio (7,9 frente a 9,6) y de conductividad media (64,3 μS frente a 202,1 μS) respectivamente. Solo se encontraron dos ejemplares de *Bulinus camerunesis* que liberaban larvas cercarias del Schistosoma en la isla central de Barombi Kotto, si bien más adelante se detectó ADN del Schistosoma en muestras de *Bulinus* de ambos lagos, así como en *Indoplanorbis exustus*, una especie invasora de origen asiático.

Conclusiones: Los niveles actuales de helmintiasis transmitida por el suelo son muy bajos, mientras que la esquistosomiasis urogenital es más preocupante en Barombi Kotto. Esta evaluación pone de manifiesto una oportunidad única de realizar más estudios acerca de la dinámica epidemiológica de estos lagos de cráter para evaluar la posible intensificación de las intervenciones, tanto destinadas a incrementar y mantener el control en Barombi Kotto como a frenar la transmisión de ambas enfermedades en Barombi Mbo.

Translated from English version into Spanish by Paloma Muñoyerro, through

TRANSLATORS
WITHOUT BORDERS
